# Painful knee joint after ACL reconstruction using biodegradable interference screws- SPECT/CT a valuable diagnostic tool? A case report

**DOI:** 10.1186/1758-2555-2-24

**Published:** 2010-09-16

**Authors:** Michael T Hirschmann, Tom Adler, Helmut Rasch, Rolf W Hügli, Niklaus F Friederich, Markus P Arnold

**Affiliations:** 1 Department of Orthopaedic Surgery and Traumatology, Kantonsspital Bruderholz, CH-4101 Bruderholz, Switzerland; 2 Institute for Radiology and Nuclear Medicine, Kantonsspital Bruderholz, CH-4101 Bruderholz, Switzerland

## Abstract

With the presented case we strive to introduce combined single photon emission computerized tomography and conventional computer tomography (SPECT/CT) as new diagnostic imaging modality and illustrate the possible clinical value in patients after ACL reconstruction. We report the case of a painful knee due to a foreign body reaction and delayed degradation of the biodegradable interference screws after ACL reconstruction. The MRI showed an intact ACL graft, a possible tibial cyclops lesion and a patella infera. There was no increased fluid collection within the bone tunnels. The 99mTc-HDP-SPECT/CT clearly identified a highly increased tracer uptake around and within the tibial and femoral tunnels and the patellofemoral joint. On 3D-CT out of the SPECT/CT data the femoral graft attachment was shallow (50% along the Blumensaat's line) and high in the notch. At revision arthroscopy a diffuse hypertrophy of the synovium, scarring of the Hoffa fat pad and a cyclops lesion of the former ACL graft was found. The interference screws were partially degraded and under palpation and pressure a grey fluid-like substance drained into the joint. The interference screws and the ACL graft were removed and an arthrolysis performed.

In the case presented it was most likely a combination of improper graft placement, delayed degradation of the interference screws and unknown biological factors. The too shallow and high ACL graft placement might have led to roof impingement, chronic intraarticular inflammation and hence the delayed degradation of the screws.

SPECT/CT has facilitated the establishment of diagnosis, process of decision making and further treatment in patients with knee pain after ACL reconstruction. From the combination of structural (tunnel position in 3D-CT) and metabolic information (tracer uptake in SPECT/CT) the patient's cause of the pain was established.

## Background

In the last decades interest among arthroscopic surgeons in using biodegradable interference screws has grown [1-12]. Biodegradable interference screws are either made from poly-lactid-acid-PLA or polyglycolide-PGA (e.g. poly-L-lactide acid (PLLA) or poly-DL-lactide acid (PDLLA)) alone or in composite with hydroxy-apatite and tricalcium-phosphate [1,3,5,11,13]. Offering similar fixation strength and outcome as metal interference screws these promise to cause less graft damage at insertion, less artifacts in magnetic resonance imaging and generally no removal of the screws is necessary [14]. However, the evidence on the use of biodegradable interference screws in anterior cruciate ligament (ACL) reconstruction is still scarce. Although the screws promise to degrade within approximately 12-24 months after surgery, several cases have been reported in which this did not happen [1,10,15].

Also a variety of other screw related problems such as screw breakage, intraarticular migration, allergic or foreign body reactions, arthrosynovitis, cyst and abscess formation, fibroxanthoma and tunnel enlargement have been reported [1,10,11,13,16-20].

In most orthopaedic units current diagnostic follow-up in patients having problems after ACL reconstruction include clinical examination, standardized radiographs, computerized tomography (CT), magnetic resonance imaging (MRI) [21-23]. Clinical examination is often not specific enough and radiographs are only able to detect gross structural abnormalities, therefore these often fail to identify the origin of the patient's pain. MRI is a sensitive imaging modality, but it has been reported that persistence of biodegradable interference screws was seen although it already had been degraded [7]. SPECT/CT combines the precise anatomical detail available with high spatial resolution CT and information on abnormal bone metabolism through single photon emission computerized tomography (SPECT) [24-28]. It promises the potential assessment of the biology of the joint and particularly the bone-graft-fixation complex [28].

To the best of our knowledge there is no case report or study in the literature concerning the use of SPECT/CT in patients with pain after ACL reconstruction. With the case described we strive to introduce SPECT/CT as new diagnostic imaging modality and illustrate the possible clinical value in patients after ACL reconstruction it may offer as new diagnostic radiologic modality.

## Case report

A 29 year old woman sustained a valgus-hyperflexion injury of her left knee while skiing 2 years ago. An ACL intrasubstance tear and a radial lesion of the lateral meniscus were diagnosed and treated by an arthroscopically assisted single-bundle ACL reconstruction using a bone-patellar tendon- bone (BTB) autograft three months after injury. The graft was fixed within the femoral and tibial tunnel using PLGA/TCP interference screws (Milagro™, Depuy MITEK, Spreitenbach, Switzerland). The Milagro™ interference screw is made of 30% β-TCP (tricalcium phosphate) and 70% PLGA (polylactic-co-glycolic acid). In addition, a partial lateral meniscectomy was performed.

On the contralateral knee an ACL reconstruction using patellar tendon autograft was performed 8 years before. At this time the graft was fixed within the tibial and femoral tunnels using sutures around post screws.

**Figure 1:**
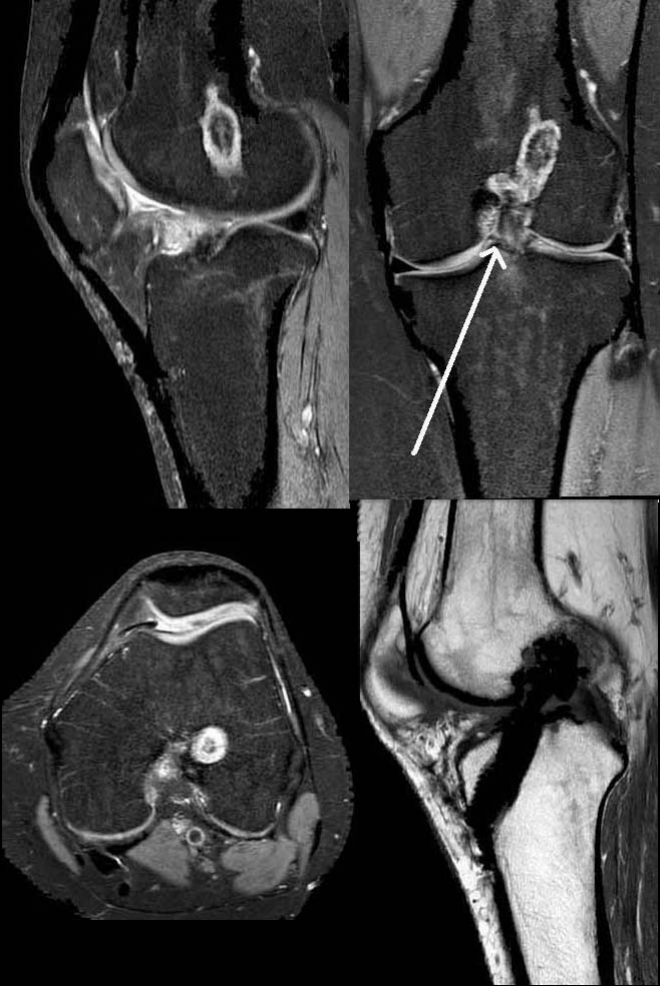
**The MRI (PDW FS sagittal, PDW FS coronal, PDW FS axial, T1 TSE sagittal) showed an intact continuity of the ACL graft and a possible tibial cyclops lesion**.

6 months after initial surgery the patient underwent a revision arthroscopy with arthrolysis of the patellar ligament and Hoffa's fat pad due to pain and limited flexion, which was attributed to scarring of the anterior joint interval and a patella infera. The patient's problems did not resolve. Twelve months later at initial presentation to our clinic she still presented with a limited flexion (<95°) and a patella infera. Climbing stairs caused excruciating anterior knee pain and the knee felt unstable. Lachman and Slocum test showed a slight increase of anterior-posterior translation in relation to the contralateral knee. A firm endpoint was present. The MRI showed an intact ACL graft, a possible tibial cyclops lesion and a patella infera. There was no increased fluid collection within the bone tunnels (fig. 1).

**Figure 2:**
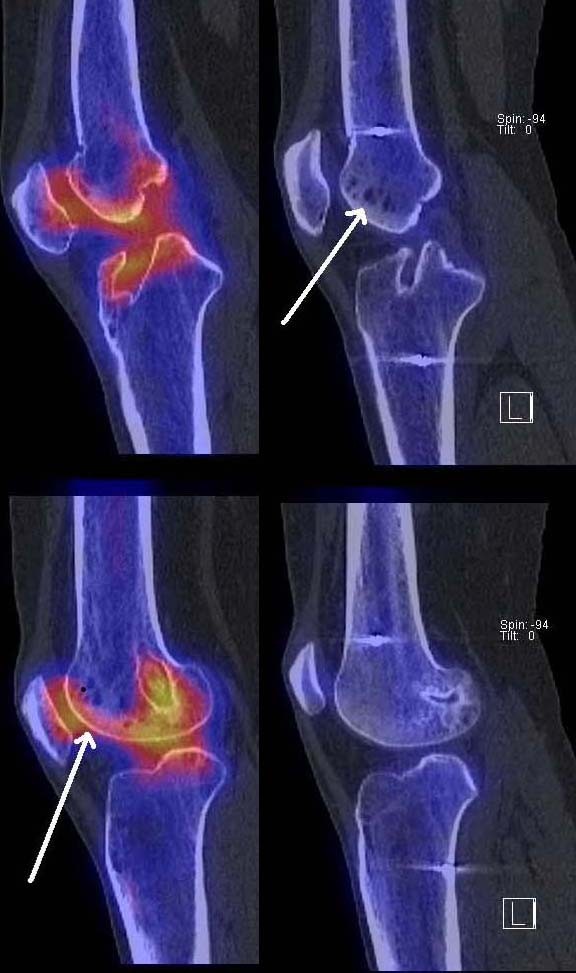
**Sagittal, axial and coronal reformation of right and left knee in 99mTc-HDP-SPECT/CT showing increased tracer uptake within the femoral and tibial tunnel and patellofemoral joint of the left knee**. The right knee did not demonstrate any areas of increased tracer uptake.

**Figure 3:**
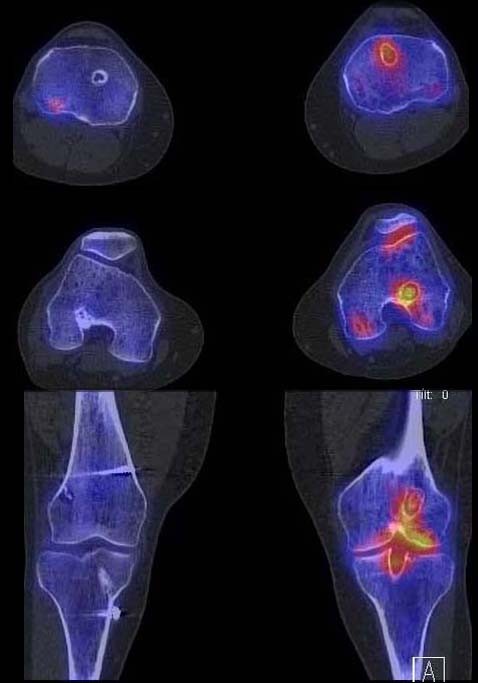
**Sagittal, axial and coronal reformation of right and left knee in 99mTc-HDP-SPECT/CT showing increased tracer uptake within the femoral and tibial tunnel and patellofemoral joint of the left knee**. The right knee did not demonstrate any areas of increased tracer uptake.

**Figure 4:**
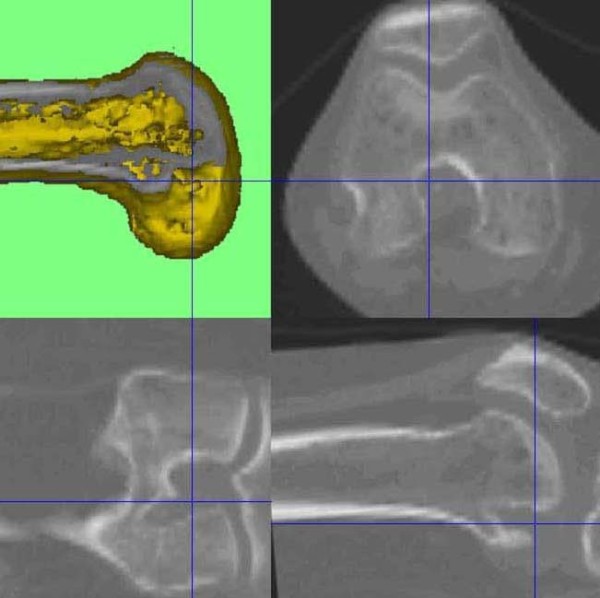
**CT out of the SPECT/CT data showing femoral and tibial bone tunnels 3D-CT analysis, which indicate a shallow and high placed ACL graft on the left knee**.

**Figure 5:**
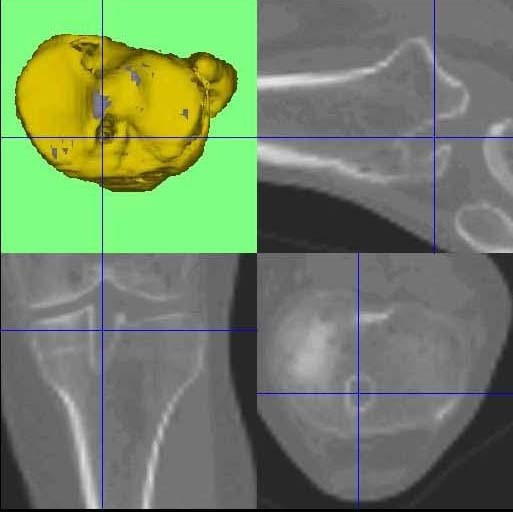
**CT out of the SPECT/CT data showing femoral and tibial bone tunnels 3D-CT analysis, which indicate a shallow and high placed ACL graft on the left knee**.

**Figure 6:**
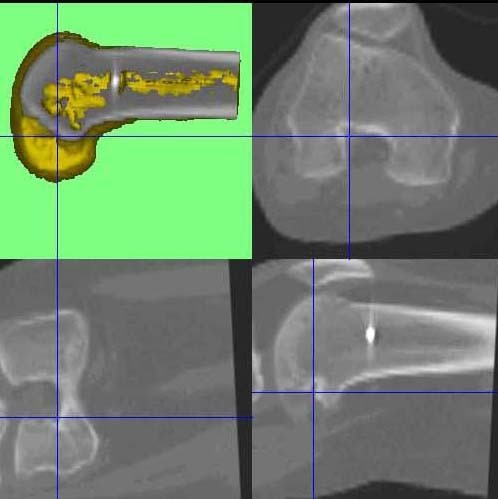
**On the right knee the femoral tunnel is placed far more posterior**. The tibial tunnels indicate a proper graft placement.

**Figure 7:**
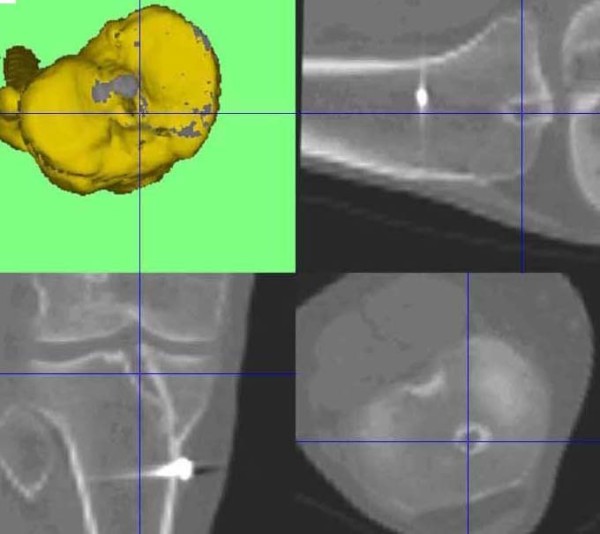
**On the right knee the femoral tunnel is placed far more posterior**. The tibial tunnels indicate a proper graft placement.

The 99mTc-HDP-SPECT/CT clearly identified a highly increased tracer uptake around and within the tibial and femoral tunnels and the patellofemoral joint (fig. 2, fig. 3). In addition, the femoral bone showed a diffuse patchy osteopenia. On the femoral side the graft attached at a shallow (50% along the Blumensaat's line) and high position according to Amis et al. (fig. 4, fig. 5, fig 6, fig. 7) [29]. On the contralateral side the graft complex, the tibial and femoral tunnels showed no increased tracer uptake.

At revision arthroscopy a diffuse hypertrophy of the synovium, scarring of the Hoffa fat pad and a cyclops lesion of the former ACL graft was found causing graft to notch roof impingement in extension and a substantial flexion deficit. The interference screws were partially degraded and under palpation and pressure a grey fluid-like substance drained into the joint.

**Figure 8:**
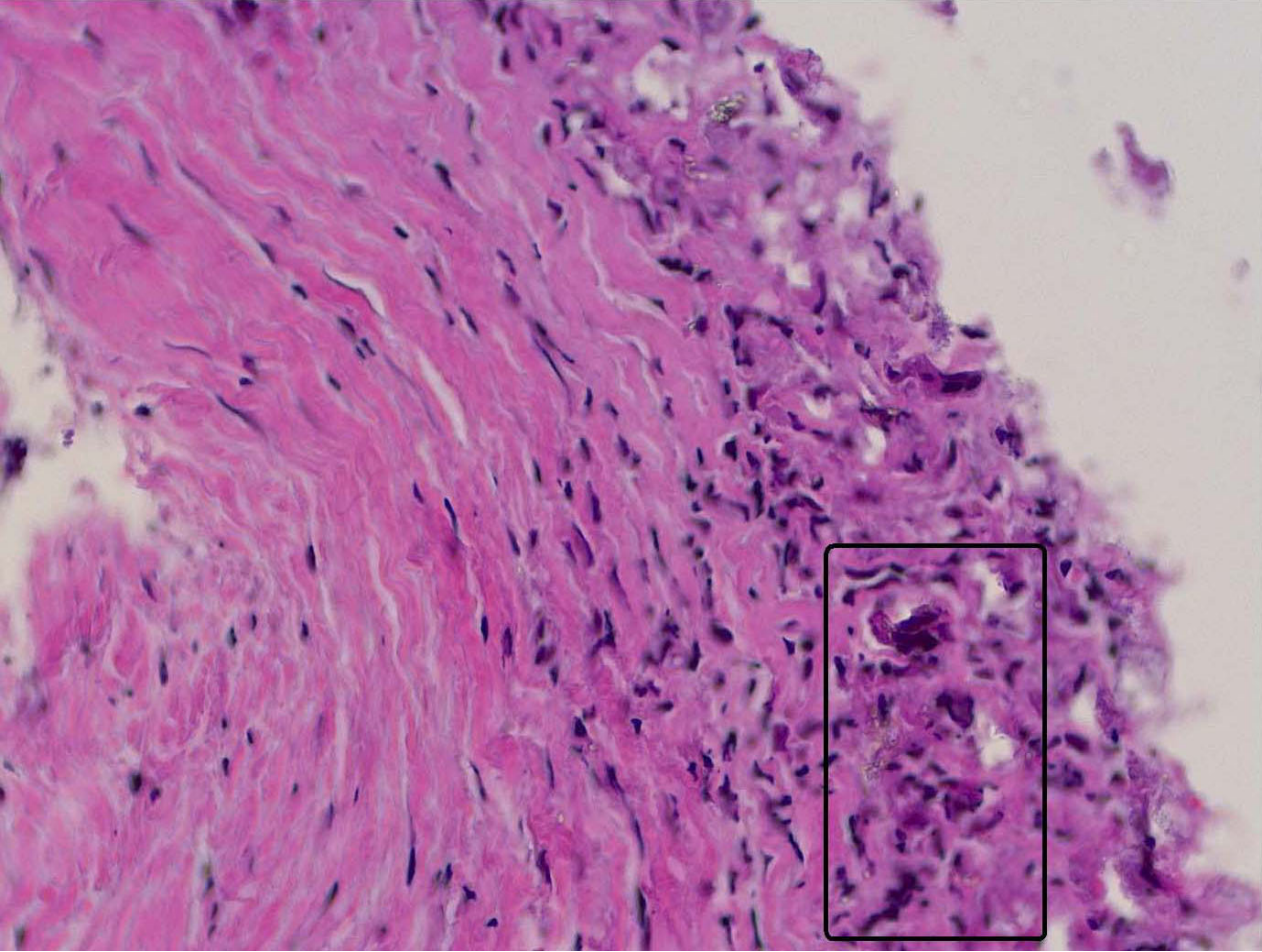
**Histopathological examination using hematoxylin&eosin of the degraded screw material showing foreign body reaction**.

The Milagro™ interference screws were removed, we performed a debridement of the ACL graft and an arthrolysis, in particular of the anterior patellar ligament interval. The histological examination (fig. 8) of the degraded screw material and removed fibrous tissue showed typical findings of a foreign body reaction. Microbiologic specimens were normal. Intraoperatively full extension and flexion of 135° was regained. The patient fully recovered from surgery and was pain free as she was with her right knee, which had been undergone ACL reconstruction using post screw graft fixation.

## Discussion

The most important finding of the present case was twofold:

Firstly, we reported the case of a painful knee due to a foreign body reaction and degradation under pressure after ACL graft fixation using biodegradable interference screws. According to Frosch et al. 18 months after ACL reconstruction one could expect that the used interference screws were entirely degraded and partially incorporated [1,10,15]. The authors investigated the in vivo degradation and biocompatibility of the same interference screw as in our case report. In a series of 12 patients after arthroscopic ACL reconstruction using hamstring tendon grafts serial MR imaging was performed at 3, 6 and 12 months after surgery [1,10,15]. At 12 months post-operatively the average volume loss of the tibial and femoral screws were 83 ± 17% and 92 ± 6%, respectively [1,10,15]. The screws were entirely degraded and only remants were identifiable [1,10,15]. In contrast, Ma et al. found no full degradation of PLLA interference screws at at 2-4 years after surgery in patients after hamstring ACL reconstruction [30]. Fink et al. showed similar results with complete degradation at 12 months using CT scans on PGA screws [11]

Although the Milagro™ screw is considered to be biocompatible and osteoconductive the femoral and to a lesser degree the tibial screw in the presented case did not degrade and caused a foreign body reaction, which was confimed by histology. This is in line with others describing inflammatory or foreign body reactions after use of biodegradable screws [1,7,10,13]. In which patients and under which condition and environment the degradation of this biodegradable interference screw causes a foreign body reaction and related arthrofibrosis remains unclear. A number of different factors such as the polymers and copolymers, the size of the screw, the position of the interference screw and the sterilization are considered to be key factors for the degradation of biodegradable screws [1]. Also the angulation of the tunnel, location of tunnel entrance, the angle and depth of graft fixation and the joint metabolism might be of influence not only for the degradation process but also for the graft tension [21,31,32].

In the case presented it seems to be a combination of improper graft placement, delayed degradation of the used interference screws and biological factors, which could not be elucidated. The too shallow and high graft placement might have led to impingement and thus chronic intraarticular inflammation.

Secondly SPECT/CT may facilitate the establishment of diagnosis, process of decision making and further treatment in patients with knee pain after ACL reconstruction. Due to the combination of structural, functional and metabolic information the patient's cause of the pain was established [25,26].

Marchant et al. investigated the intra- and inter-observer reliability in identifying bone tunnels and assessing tunnel widening on radiographs, MRI and CT [21]. They showed that CT was most reliable. Radiographs and MRI were not reliable, even for simply identifying the presence of a bone tunnel [21]. Without the use of SPECT/CT the decision to debride the ACL graft and remove the interference screws would have not been made.

Three dimensional reconstructions of CT provide the surgeon with a better 3D understanding of patient's anatomy [22]. The position of the tunnels, the orientation of the graft and the localization of attachment area can be assessed, which have been reported to be decisive for outcome after ACL reconstruction [33,34]. In the presented case 3D-CT out of the SPECT/CT data was used to accurately evaluate the tunnel placement in relation to standardized frames of reference.

Hogervorst et al. found that scintigraphic tracer uptake correlated significantly with tibial tunnel enlargement and location of the tibial tunnel [35]. They concluded that bone scintigraphy is a reliable method assessing the complex biology of graft remodelling, tunnel enlargement and osseous homeostasis [35]. In contrast to scintigraphy or SPECT alone SPECT/CT is able to accurately allocate the tracer uptake to anatomical areas (e.g. tibiofemoral or patellofemoral joint as well as tibial and femoral tunnels).

## Consent

Written informed consent was obtained from the patient for publication of this case report and any accompanying images.

## Competing interests

The authors declare that they have no competing interests.

## Authors' contributions

MTH, TA, HR, RWH, NFF and MPA put together the patient's case report, drafted the manuscript and performed a review of the current literature. All authors read and approved the final manuscript.
